# Athletic identity and sport injury: a systematic review and meta-aggregation

**DOI:** 10.1186/s40359-025-03902-7

**Published:** 2025-12-23

**Authors:** Siqi Liu, Young-Eun Noh, Jeonghyo Kim

**Affiliations:** 1 Faculty of Sports and Exercise Science, Universiti Malaya, Kuala Lumpur, 50603 Malaysia; 2 Department of Physical Education, Seoul National University, Seoul, 08826 South Korea

**Keywords:** Psychological response, Injured athlete, Athletic rehabilitation, Post-injury adaptation, Self-concept, Resilience

## Abstract

**Objective:**

This review aimed to systematically review qualitative research examining changes in athletic identity following sports injuries.

**Methods:**

The review was conducted in accordance with the Preferred Reporting Items for Systematic Reviews and Meta-Analyses (PRISMA 2020) guidelines. Electronic databases searched included Web of Science, Psychology & Behavioral Sciences Collection, PubMed, SPORTDiscus, and Scopus. Manual searches were conducted using forward and backward citation tracking via Google Scholar. Study quality was assessed using the Mixed Methods Appraisal Tool. Data extraction and synthesis followed the Joanna Briggs Institute guidelines, and the ConQual approach was used to evaluate the confidence level of findings based on dependability and credibility.

**Results:**

A total of 24 qualitative studies were included. Using a meta-aggregative approach, findings were synthesized into two overarching themes: “athletic identity disruption” (*n* = 17) and “athletic identity reconstruction” (*n* = 7). The theme of athletic identity disruption highlighted that sports injuries often lead to a diminished sense of athletic identity due to factors such as physical limitations, withdrawal from competition, and psychological distress. In contrast, the athletic identity reconstruction theme showed that some athletes rebuild their athletic identity by engaging in alternative roles, such as coaching, volunteering, or participating in new sports.

**Conclusion:**

Sports injuries can have profound and varied effects on athletic identity. While some athletes experience disruption and identity loss, others successfully reconstruct their sense of self within the sporting context. Future research should consider more diverse study designs, such as longitudinal and mixed-methods studies, and further investigate how cultural, social, and contextual factors influence athletes’ experiences of identity disruption and reconstruction.

**Supplementary Information:**

The online version contains supplementary material available at 10.1186/s40359-025-03902-7.

Identity has long been recognized as a central construct in psychology and sociology, shaping how individuals relate to themselves and others through a dynamic process that is associated with both social context and personal agency. Social identity has been defined as “that part of an individual’s self-concept which derives from his/her knowledge of his/her membership of a social group (or groups) together with the value and emotional significance attached to that membership” (p. 255) [1]. Some social identities are predetermined at birth (e.g., identities related to nationality or race). In contrast, others (e.g., athletic identity) are developed (or acquired) through an individual’s conscious choices and investment in activities [2, 3]. Athletic identity is defined as “the degree to which an individual identifies with the athlete role” (p. 237) [4]. Conceptually, while social identity emphasizes group membership and the associated social and emotional significance, athletic identity focuses on the personal meaning of being an athlete and the extent to which this role influences one’s self-concept, behaviors, and daily life. Thus, athletic identity can be seen as a specific facet of social identity, distinguished by its behavioral foundation and role-centered psychological impact.

Athletic identity is a central construct that shapes how athletes think, feel, and behave both within and beyond the sporting environment. An athlete’s self-perception carries far-reaching implications for motivation, performance, coping strategies, and long-term psychological adjustment. Research demonstrates that athletic identity is associated with a wide range of psychological and behavioral factors, including moral disengagement in doping [5], nutritional habits [6], receptiveness to coaching feedback [7], gender role conflict [8], exercise addiction [9], stress levels [10], intentions regarding substance use [11], concussion reporting behaviors [12], and vulnerability to sports injuries [13]. Notably, many athletes continue to maintain a strong athletic identity even after retirement [14]. This enduring identity may persist throughout adulthood [15, 16] and has been linked to mental health outcomes in retirement [17] as well as broader aspects of long-term well-being [18].

Recent research on athletic identity has focused on the theme of the COVID-19 pandemic. The pandemic disrupted athletes’ competitions and training routines, posing challenges to their professional careers [19] and adversely impacting their mental health, with reported increases in psychological distress, depression, and anxiety [20]. During this period, studies found that although the level of athletic identity was not associated with athletes’ confidence in returning to sports after the pandemic [21], it was related to their emotional and cognitive regulation strategies [22] and mental health [23], including the incidence of depression [13] and levels of anxiety [24]. Interestingly, an interview with 27 Paralympic athletes found that, even during the pandemic lockdown, athletes maintained a high level of athletic identity [2]. Moreover, a survey involving 234 student-athletes found that 28.89% of participants reported an increase in their athletic identity level during the pandemic lockdown compared to before the pandemic [25].

For this review, sport injury is defined as bodily harm or acquired physical impairment-whether sustained during sport participation [26] or through non-sport-related events-that results in temporary or permanent disruption to sport participation, athletic functioning, or self-identification as an athlete. When athletes experience interruptions to competition and training due to sports injuries, their athletic identity may be challenged [27–30]. To better understand how injury challenges athletic identity, it is useful to distinguish between the broader notion of identity loss and the more specific loss of athletic identity. Identity loss refers to a lack of clarity about one’s sense of self as a person, often producing existential distress, whereas loss of athletic identity specifically pertains to a reduction in identification with the athlete role or the inability to enact a role to which one remains strongly attached. In the context of sport injury, the distress experienced by athletes may arise from either not knowing who they are as individuals or from being unable to fulfill the athletic role central to their self-concept. Building on this, Aston et al. [14] introduced the related concepts of identity gripping and identity flight. Identity gripping occurs when an individual’s identity becomes tightly held and resistant to change, potentially exacerbating stress when that identity is challenged, while identity flight describes a tendency to disengage or distance oneself from a threatened identity, representing a coping or protective mechanism. These frameworks provide a nuanced perspective on the ways athletes navigate identity-related challenges following injury. The disruption of athletic identity can be associated with their emotional responses and adherence during rehabilitation [31], as well as influence their willingness to self-medicate [32], perceived pain levels [33], intention to return to sport [34], post-injury athletic performance [35], and psychological readiness to return to sport [36, 37].

A recent scoping review identified five key themes related to athletic identity and response to sports injuries, based on an analysis of 22 quantitative studies [38]. These themes encompassed psychosocial, behavioral, and injury-related outcomes, along with demographic factors and pain. Building on this work, Brewer and Chatterton [39] conducted a supplementary narrative review that summarized quantitative research from the past five years on the role of athletic identity in injury responses and outcomes. Their review highlighted the associations between athletic identity and symptoms of depression and concussion, coping strategies, fear-avoidance beliefs, rehabilitation adherence, post-return athletic performance, and psychological readiness.

However, both reviews exclusively included quantitative studies that used the Athletic Identity Measurement Scale (AIMS [4]). Although AIMS remains the most widely used tool for measuring athletic identity in research [40, 41], it is important to recognize that its sensitivity may vary across populations, particularly among athletes at different levels of sports participation [42]. As such, conclusions based solely on AIMS may not fully reflect the complexities of athletic identity in real-world contexts.

Recognizing the limitations of the original AIMS, Brewer et al. [43] recently introduced the Athletic Identity Measurement Scale-Third Generation (AIMS-3G), which retains the core construct of athletic identity while expanding the framework to include two components: athletic identity attributes and athletic identity processes. The attributes component refers to descriptive features of athletic identity, such as prominence and self-worth contingency, while the processes component captures social and behavioral aspects, including social reinforcement and self-presentation [44]. This expanded framework provides a more comprehensive approach for assessing athletic identity [43].

However, in Renton et al.’s scoping review [38], one sub-theme explored the relationship between injury as an exposure and athletic identity as an outcome. Of the three studies included in this category [45–47], only one [46] assessed athletic identity both before and after injury, reporting small, nonsignificant declines. Based on this limited evidence, Renton et al. [37] concluded that there is insufficient support to define a clear relationship between injury and changes in athletic identity. Similarly, a recent historical cohort study found no significant difference in athletic identity levels between athletes with a history of ankle sprains and a control group, even 3 to 15 years post-injury [42].

In light of these limitations, Brewer and Chatterton [39] recommended further exploration of qualitative data to better understand the core role of athletic identity in the context of sports injury. Qualitative research, in contrast to variable-driven approaches, seeks to uncover how individuals make meaning of their experiences [48], with the researcher serving as the primary instrument for data interpretation [49]. It offers context-sensitive insights, deepens theoretical development, and provides rich, in-depth perspectives on individuals and groups [50]. Despite these strengths, a synthesis of qualitative findings related to athletic identity and sport injury remains lacking. This gap leads to the guiding question of the present systematic review: How is athletic identity experienced following sports injury from a qualitative research perspective?

Meta-aggregation is a recognized method for synthesizing qualitative research evidence [51, 52]. A key feature of this approach is that reviewers refrain from reinterpreting the findings of the original studies; instead, they present the results as reported by the primary authors [53]. Similar to quantitative systematic reviews, which are characterized by methodological rigor and transparency, the meta-aggregative approach follows a structured and systematic process for integrating qualitative findings. Therefore, it is more closely aligned with the principles of systematic reviewing than many other qualitative synthesis methods [52]. Importantly, the meta-aggregation can accommodate heterogeneity across qualitative studies by focusing on the authors’ analysis and interpretation of their data. This allows for the aggregation of findings from studies that explore the same phenomenon of interest, regardless of methodological orientation (e.g., phenomenology, ethnography, and grounded theory) [51]. This method has been applied in qualitative systematic reviews on topics such as sport injury experiences [54] and the effectiveness of Sport Injury Prevention Programmes (SIPPs) [55]. Based on this approach, one of the two studies synthesized and categorized the factors influencing the implementation of SIPPs [54]. Another meta-aggregative study summarized that adolescents experience a range of positive and negative emotions throughout the injury-recovery process [55]. These studies demonstrate the effectiveness of the meta-aggregation approach in synthesizing qualitative research findings. Therefore, in the present study, we adopted meta-aggregation to integrate the existing qualitative evidence on the relationship between athletic identity and sport injury within the context of athletic injury.

This review addresses a critical gap in the literature by synthesizing qualitative evidence and providing in-depth insights into athletes’ subjective experiences, coping strategies, and identity negotiation processes. The findings have important practical implications for coaches, sport psychologists, healthcare professionals, and policymakers seeking to support athletes’ psychological well-being and optimize post-injury outcomes. For example, a clearer understanding of how athletes perceive and reshape their athletic identity after injury can inform the development of targeted rehabilitation strategies, enhance mental health support, and facilitate a successful return to sport. Therefore, this review is both necessary and timely, as it advances theoretical understanding while offering valuable guidance for applied practice in sport contexts.

## Methods

### Search strategy

This systematic review has been registered in PROSPERO (registration number: CRD420251105079). This review followed the Preferred Reporting Items for Systematic Reviews and Meta-Analyses (PRISMA 2020) statement [56] and employed both electronic database searching and manual searching. The electronic database search included Web of Science, Psychology & Behavioral Sciences Collection, PubMed, SPORTDiscus, and Scopus. The first author developed search keywords based on the terms used in a topic-related scope [38] and a supplementary narrative review [39]. The search keywords covered the three main themes of athlete, identity, and injury. Specifically, the keywords were: (athlete* OR Paralympian OR Olympian) AND (identit*) AND (injur* OR wound* OR tear OR sprain OR concussion OR fracture*). No date restrictions were applied during the search process. The final search date was November 20, 2024. The manual searching involved both forward and backward citation tracking through the Google Scholar search engine, and the results were stored in Zotero (2016) for further screening. The manual searching included: (1) articles that cited AIMS and AIMS-3G, (2) articles cited in the included studies identified via the electronic database searching, and (3) reviews related to the biopsychosocial responses to sports injuries [57] and reviews related to the athletic identity [38, 39].

### Eligibility criteria

The inclusion criteria for this review comprised two key elements: (1) articles published in international journals in English; (2) articles that were qualitative research, including but not limited to designs. The exclusion criteria included the following: (1) articles unrelated to athletic identity topics, such as social identity, as athletic identity is only a part of social identity [2]; (2) articles unrelated to injury or where the injury involves something other than the athlete (e.g., their horse); (3) grey literature; (4) review studies, quantitative studies, and mixed-methods research.

### Literature screening

The literature screening involved four steps: (1) duplicate removal, (2) primary screening, (3) secondary screening, and (4) expert consensus. The duplicate removal was carried out in Zotero (2016). The primary screening process included the independent assessment of titles and abstracts. The secondary screening involved screening the included full-text literature based on the established inclusion and exclusion criteria. Expert consensus was reached through deliberations among all the authors, engaging in discussions with an expert who has publication experience in international sports psychology journals. After this process, a study [58] examining the impact of horse injuries on equestrian athletes’ athletic identity was excluded, as this review focuses solely on athlete injuries.

### Quality assessment

The methodological quality appraisal utilized the Mixed Methods Appraisal Tool (MMAT) [59]. This tool is a building block that includes five types of research designs, with one category specifically for assessing the methodological quality of qualitative research designs through five core criteria (e.g., “Is the qualitative approach appropriate to answer the research question?”). Each criterion includes three options: “Yes,” “No,” or “Can’t Tell.”

### Data extraction and synthesis

We used a table created in Microsoft Excel to extract data from the selected studies, including study author(s), publication year, aim, methodology, data collection, data analysis, country, participants, types of sports, sports levels, injury experience, number of participants, gender, age, and conclusion. This study followed the Joanna Briggs Institute (JBI) guidelines [52] for evidence synthesis. The process involves three steps: *Finding*, *Category*, *and Synthesized Finding*. (1) Finding: This step involves a verbatim extract of the included study’s analytic interpretation of their results or data, which are then presented in the meta-aggregation table as findings. Each finding is accompanied by illustrations, which are direct quotations from participants, fieldwork observations, or other supporting data from the paper. (2) Category: This step involves the aggregation and explanatory statements of multiple similar findings, forming a conceptual summary. (3) Synthesized Finding: The final step is an overarching description of a group of categorized findings, providing a comprehensive interpretation of the grouped findings.

### Risk of bias and publication bias

Since this review is a qualitative synthesis, statistical methods were not applied to assess risk of bias or publication bias. Instead, potential biases were addressed through methodological quality appraisal, confidence assessment, an independent dual-review process, author discussions, and consultation with subject-matter experts to ensure the credibility and dependability of the included studies.

### Confidence assessment

This review used the ConQual method [53] to assess confidence in meta-aggregated findings. This method assesses the dependability of meta-aggregated results through a checklist containing five “yes” or “no” questions (e.g., “Is there congruity between the research methodology and the research question or objectives?”). For evaluating the credibility of the findings, the ConQual method uses three ranking criteria: *Unequivocal*,* Credible*,* and Not Supported.* Unequivocal findings are considered beyond reasonable doubt, Credible findings are plausible in light of data, which can be logically inferred from the data, and Not Supported findings are not backed by data; therefore, should not be included in the meta-aggregative process. Based on these two evaluations, the overall ranking of the qualitative study results can be classified into four levels: high, moderate, low, and very low. ConQual assumes that the initial confidence level of all studies is considered “high.” After the dependability assessment, the confidence level of each study may decrease. Specifically, if the dependability assessment results in 4–5 “yes” responses, the confidence level remains unchanged; 2–3 “yes” responses lead to a one-level downgrade, and 0–1 “yes” responses lead to a two-level downgrade. After the credibility evaluation, if the assessment results include both unequivocal and credible findings, the synthesized finding can be downgraded by one level (−1). For credible findings, the synthesized finding can be downgraded by two levels (−2). If both credible and unsupported findings are present, it can be downgraded by three levels (−3), and unsupported findings alone result in a four-level downgrade (−4).

### Independent dual review process

In this review, the manual and electronic database searches, literature screening (excluding expert consensus), quality assessment, data extraction and synthesis, and confidence assessment were independently conducted by the first and second author of this study. Any discrepancies in data extraction were first discussed between the two primary reviewers. When consensus could not be reached, the third reviewer was consulted to independently evaluate the extracted information and provide an adjudicating decision.

## Results

**Fig. 1:**
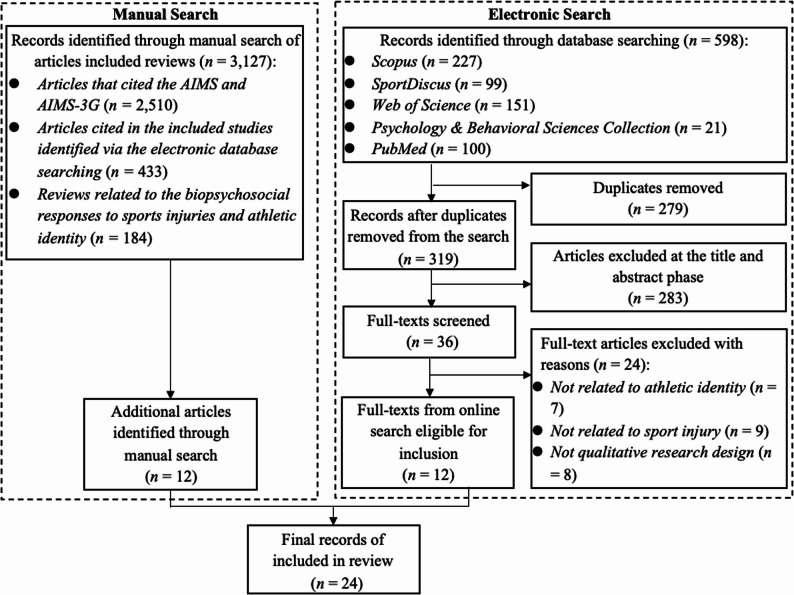
The PRISMA flow diagram

The electronic search yielded a total of 598 articles. Following the screening process, 12 articles were included. A manual search yielded 12 more articles; therefore, a total of 24 articles were included in this review (see Fig. 1). Based on the JBI guidelines for evidence synthesis [52], these included papers were synthesized into two themes: (1) athletic identity disruption (*n* = 17) and (2) athletic identity reconstruction (*n* = 7).

### Quality assessment

 A detailed breakdown of the quality assessment for each study is presented in Table 1.

**Table 1:** MMAT quality appraisal of studies (Alphabetical Order)

Author(s)	Screening Questions^*^		Qualitative^†^					Comments
	Q1	Q2	Q1	Q2	Q3	Q4	Q5	
Borg et al., 2021 [60]	✓	✓	✓	✓	✓	✓	✓	This study meets all the criteria for MMAT assessment.
Caron et al., 2017 [61]	✓	✓	✓	✓	✓	✓	✓	This study meets all the criteria for MMAT assessment.
Caron et al., 2021 [81]	✓	✓	✓	✓	✓	–	✓	This research was only able to conduct one interview with each participant. Interviews with teammates and coaches were relatively short (*Mean* = 25 min).
Cassilo and Sanderson, 2019 [62]	✓	✓	✓	–	✓	✓	✓	The sample was limited to those who chose to disclose online, and not all athletes chose this option to talk about concussion experiences.
Crawford et al., 2014 [77]	✓	✓	✓	–	✓	✓	✓	This study had a small sample.
Dean, 2018	✓	✓	✓	–	✓	✓	✓	Since the data mainly came from personal experiences, the research findings may be limited in terms of the scope and depth of interpretation.
Douglas et al., 2024 [64]	✓	✓	✓	✓	–	✓	✓	All student-athletes from this study were from geographically similar locations, which could have influenced the variability within student-athlete experiences in all aspects.
Ezzat et al., 2018 [75]	✓	✓	✓	✓	✓	✓	✓	This study meets all the criteria for MMAT assessment.
Hammer et al., 2019 [73]	✓	✓	✓	✓	✓	✓	✓	This study meets all the criteria for MMAT assessment.
Hänninen and Pohjola, 2023 [65]	✓	✓	✓	–	✓	✓	✓	The data are based on only two autobiographical books; they are by no means representative of all individuals suffering sports injuries.
Hawkins et al., 2014 [71]	✓	✓	✓	–	✓	✓	✓	This study had a small sample, which only consisted of elite badminton players with a spinal cord injury.
Hockey, 2005 [76]	✓	✓	✓	✓	✓	✓	✓	This study meets all the criteria for MMAT assessment.
Karlström et al., 2022 [66]	✓	✓	✓	✓	✓	✓	✓	This study meets all the criteria for MMAT assessment.
Kavanagh, 2012 [78]	✓	✓	✓	–	✓	✓	✓	The study is based on the experience of a single individual.
Lisee et al., 2020 [67]	✓	✓	✓	–	–	✓	✓	A total of 21 interviews were conducted by a female interviewer, which may have affected the gender-based responses from male and female participants. Most patients in this study were minors and accompanied by their parents or guardians during the interviews, which may have caused them to unconsciously alter their responses.
Little et al., 2023 [68]	✓	✓	✓	✓	✓	✓	✓	This study meets all the criteria for MMAT assessment.
Murray et al., 2022 [74]	✓	✓	✓	–	✓	✓	✓	As the interviews were conducted over Skype, this may have limited the intimacy that would have been gained from in-person interviews.
Perrier et al., 2014 [79]	✓	✓	✓	✓	✓	✓	✓	This study meets all the criteria for MMAT assessment.
Seguin and Culver, 2022 [31]	✓	✓	✓	–	✓	✓	✓	The study is based on the experience of a single individual.
Sparkes and Smith, 2002 [72]	✓	✓	✓	✓	✓	✓	✓	This study meets all the criteria for MMAT assessment.
Watkins et al., 2020 [30]	✓	✓	✓	–	–	–	✓	The sample is unlikely to be representative of all people who have experienced knee injury. Knee injury pathology and associated symptoms are heterogeneous by their very nature. There is recall bias.
Zavattaro, 2014 [69]	✓	✓	✓	✓	–	✓	✓	The generalizability of the research findings may be limited, as the study relies on the self-reported experiences of individual participants.
Zurek et al., 2022 [80]	✓	✓	✓	✓	✓	–	✓	The number of subjects is a limitation.
Zwolski et al., 2024 [70]	✓	✓	✓	–	✓	✓	✓	This work is specific to the 10 youth and young adults who participated in the interviews and may not reflect the attitudes and beliefs of all who suffer an adolescent sports-related anterior cruciate ligament injury.

MMAT = Mixed Methods Appraisal Tool; ‘✓’ means that the criterion is met. ‘x’ means that the criterion is not met.‘–’ means that there is not enough information in the paper to judge if the criterion is met or not

^*^ Screening questions 1: Are there clear research questions? Screening questions 2: Do the collected data allow addressing the research questions?

^†^ The assessment columns for four study designs (i.e., quantitative randomized controlled trials, quantitative non-randomized, quantitative descriptive, and mixed methods) have been removed because these types of study designs were not utilized in this review study. The five criteria for the qualitative studies are as follows: (1) Is the qualitative approach appropriate to answer the research question? (2) Are the qualitative data collection methods adequate to address the research question? (3) Are the findings adequately derived from the data? (4) Is the interpretation of results sufficiently substantiated by data? (5) Is there coherence between qualitative data sources, collection, analysis, and interpretation?

The main findings extracted from each study, including study design, population, data analysis, types of sport, injury experience, and key outcomes, are summarized in Table 2.

**Table 2:** The summary of findings from the included articles (Alphabetical Order)

Author(s) and Year	Aim	Methodology	Data Collection	Data Analysis	Country	Participants	Types of Sport	Sports Levels	Injury Experience	Number of Participants and Gender (Male/ Female)	Age (Year)	Conclusion(s)
Borg et al., 2021 [60]	To explore the experience of footballers who had experienced a sports-related injury	N/A	Individual semi-structured interviews	Interpretative phenomenological analysis	Malta	Athletes	Footballer (*N* = 6)	N/A	λ Legs/feet injury (*n* = 5) λ Concussions (*n* = 1)	6 (4/2)	N/A	The initial reaction to injury in athletes is often associated with changes in their athletic identity.
Caron et al., 2017 [61]	To explore a female university volleyball student athlete’s experience with protracted concussion symptoms	λ Narrative inquiry λ Deweyan pragmatic ontology	λ Visual methods λ Audiologs	λ Fluid reading λ Narrative construction λ Three-dimensional narrative inquiry space	Canada	Athlete	Volleyball (*N* = 1)	University level	Concussions	1 (0/1)	N/A	Following a concussion, athletes may experience a change or reduction in their athletic identity.
Caron et al., 2021 [81]	To explore athletes’ recovery and reintegration into the team environment following a sport-related concussion	λ Interpretivist λ Epistemology	Individual semi-structured interviews	Thematic narrative analysis	Canada	λ Athletes λ Teammates λ Coaches	λ Rugby (*n* = 1) λ Soccer (*n* = 1) λ Basketball (*n* = 1)	University level	Concussions	12	N/A	Concussion is associated with changes in athletic identity among student-athletes.
Cassilo and Sanderson, 2019 [62]	To gain a richer understanding of athletes’ lived experiences with concussions	N/A	Public online support group websites	Theory-driven deductive analysis	USA	Athletes	λ Soccer (*n* = 16) λ Others	N/A	Concussions	N/A	N/A	Concussions are associated with life alteration, social isolation, changes in relationships, and changes in athletic identity.
Crawford et al., 2014 [77]	To examine the perceptions and experiences of post traumatic growth in ParaSport athletes with acquired spinal cord injury	N/A	Individual semi-structured interviews	Phenomenological analysis	Canada	ParaSport athletes	λ Wheelchair racing λ Basketball λ Rowing λ Alpine ski racing, λ Paracycling λ Rugby λ Waterskiing λ Sledge hockey λ Baseball λ Sailing λ Tennis	λ International level λ Provincial level λ Regional level	Spinal cord injury	12	40.67 ± 9.96	Athletes who experience spinal cord injuries may maintain their athletic identity through participation in adaptive sports.
Dean, 2019 [63]	To explore how sport-related concussion can be understood within a socio-cultural context	N/A	Autoethno-graphy	Narrative analysis	Canada	Athletes	Lacrosse	University level	Concussions	1 (1/0)	N/A	Athletic identity may be associated with concussion injury.
Douglas et al., 2024 [64]	To explore experiences of high school athletes in order to understand their occupational engagement after sustaining a sports-related concussion	N/A	Individual semi-structured interviews	λ Phenomenological analysis λ Colaizzi method	USA	Athletes	λ Basketball (*n* = 2) λ Football (*n* = 3) λ Soccer (*n* = 1)	High school level	Concussions	6 (4/2)	λ 14 λ 14 λ 15 λ 17 λ 17 λ 18	Athletic identity may be related to concussion injuries in student-athletes.
Ezzat et al., 2018 [75]	To understand the influence of the injury experience on current attitudes and beliefs about physical activity and the development of post-traumatic osteoarthritis in youth and young adults 3–10 years after a sport-related knee injury	N/A	Individual semi-structured interviews	Constant comparative analysis	Canada	Youth	N/A	Moderate and vigorous physical activity minutes per week: median (range) = 75 (0–210)	λ ACL/PCL rupture (*n* = 11) λ Meniscus (*n* = 6) λ Ligament, or fracture, or patella dislocation (*n* = 3)	20 (10/10)	Median (range) = 22.3 (16.5–26.4)	After experiencing sports injuries, most youths’ athletic identities had evolved.
Hammer et al., 2019 [73]	To examine the relevance of key components of organismic valuing theory of growth through adversity in understanding posttraumatic growth amongst paratriathletes with acquired disability	N/A	Individual semi-structured interviews	Directed content analysis	USA	Athletes	Paratriathletes (*N* = 14)	λ National level λ International level	λ Brachial plexus injury λ Above/below elbow amputation λ Visual impairment λ Traumatic brain injury λ Incomplete spinal cord injury λ Above/below knee amputation	14 (8/6)	34.6 ± 5.88	Athletic identity may be related to disabling injuries in athletes.
Hänninen and Pohjola, 2023 [65]	To discuss and compare how athletes’ athletic identity is constructed in these two very different sports	N/A	Autobiogr-aphical books	Narrative analysis	Finland	Athletes	λ Ice hockey (*n* = 1) λ Freeskiing (*n* = 1)	International level	Traumatic brain injury	2 (2/0)	N/A	Traumatic brain injury is associated with changes in athletic identity.
Hawkins et al., 2014 [71]	To establish how sport, and access to an athletic identity, has been used when adjusting to a spinal cord injury	N/A	Individual semi-structured interviews	Thematic analysis	UK	Athletes	Badminton (*N* = 8)	λ National level λ Club level	Spinal cord injury	8 (6/2)	λ 22 λ 46 λ 43 λ 33 λ 42 λ 38 λ 38 λ 28	Athletic identity may be associated with spinal cord injury.
Hockey, 2005 [76]	To examine the importance of “identity work” for the maintenance of athletic identity in the face of prolonged injury, and the part that type of work played in successful athletic rehabilitation	Autoethnogr-aphy	Personal logs	Constant comparative method	UK	Athletes	Middle/long distance runners (*N* = 2)	Amateur level	λ Medial ligament damage λ Plica λ Patellar femoral damage λ Others	2 (1/1)	λ 51 λ 37	After sustaining a sports injury, the athlete was able to maintain their identity through various means.
Karlström et al., 2022 [66]	To explore individuals’ experiences of living and coping with an ACL rupture with a specific focus on experiences significant to overall life, activity in daily living and physical activity more than one year after injury	N/A	Individual semi-structured interviews	Content analysis	Sweden	ACL-injured adults	N/A	λ Pre Tegner: Median (range) = 7.5 (5) λ Current Tegner: Median (range) = 5 (6)	ACL injury	12 (4/8)	Median (range) = 32 (19–41)	An ACL injury is associated with changes in athletic identity.
Kavanagh, 2012 [78]	To explore the personal narrative of a British Paralympic wheelchair tennis player	Interpretivism	Individual semi-structured interviews	λ Narrative inquiry λ Thematic analysis	UK	Athletes	Wheelchair tennis (*N* = 1)	Elite level	Spinal cord injury	1 (0/1)	30	After sustaining a spinal cord injury that led to disability, the individual chose to continue participating in para-sports, thereby maintaining their athletic identity.
Lisee et al., 2020 [67]	To explore gender differences in psychological readiness factors of return to sport after ACLR	N/A	Individual semi-structured interviews	Deductive thematic coding analysis	USA	Athletes	λ Baseball (*n* = 1) λ Basketball (*n* = 13) λ Cheerleading (*n* = 1) λ Football (*n* = 7) λ Soccer (*n* = 1) λ Swimming (*n* = 1) λ Volleyball (*n* = 1)	High school level	ACL injury	25 (12/13)	λ Male = 16.2 ± 1.6 λ Female = 16.4 ± 1.3	There is an association between ACL injury and athletic identity among both male and female student-athletes.
Little et al., 2023 [68]	λ To explore the contextual and emotional underpinnings of fear in people 1 year after ACL injury or surgery λ To create a conceptual framework for how this cohort formed their injury beliefs by mapping the thematic results of the qualitative analysis to the common-sense model of self-regulation	Interpretive descriptive framework	Individual semi-structured interviews	Inductive coding and thematic analysis	Australia	ACL-injured adults	N/A	N/A	ACL injury	18 (5/13)	28 ± 5.86	An ACL injury is associated with changes in athletic identity.
Murray et al., 2022 [74]	To take a lifeworld perspective to explore how living with injury was meaningful to professional rugby players	N/A	Individual semi-structured interviews	Interpretative phenomenological analysis	λ UK λ Finland	Athletes	Rugby	λ International level λ Professional club level	λ Neck injury λ Broken leg injury λ Knee and ankle injury λ Shoulder injury	5 (5/0)	N/A	There is an association between sustaining a sports injury and athletic identity.
Perrier et al., 2014 [79]	To explore why athletic identity may be lost or (re)developed after acquiring a physical disability	Narrative inquiry	Individual semi-structured interviews	λ Categorical-content analysis λ Structural analysis	Canada	λ Amateur sports participants λ Athletes	λ Running λ Softball λ Skiing λ Sailing λ field	λ Amateur level λ High performance level	Spinal cord injury	11 (4/7)	40.1 (range: 28–60 years)	Among individuals with a spinal cord injury associated with disability, some report a perceived loss of athletic identity, others anticipate regaining it in the future, and some have re-established their identity as para-athletes.
Seguin and Culver, 2022 [31]	To identify intrapersonal, interpersonal, and cultural factors that influence experience and recovery after suffering concussions	λ Participatory paradigm λ Ontology λ Epistemology	λ Individual interviews λ Focus groups λ Member reflections λ Informal discussions	Thematic narrative analysis	Canada	Athletes	λ American football λ Ice hockey λ Lacrosse λ Snowboarding λ Rugby	λ World-class elite level λ Successful-elite level λ Competitive-elite level λ Semi-elite level	Concussions	12 (8/4)	N/A	Concussions are associated with changes in athletic identity among elite athletes.
Sparkes and Smith, 2002 [72]	To illustrate how body-self relationships moved from an absent presence in the lives of spinal cord injured men to something that was other, problematic, and alien	λ Interpretive interactionism λ Interpretive biography	Individual semi-structured interviews	λ Thematic analysis λ Reflexive analysis	UK	Athletes	Rugby football	λ County level λ England U19 level	Spinal cord injury	4 (4/0)	N/A	There is an association between spinal cord injury and athletic identity.
Watkins et al., 2020 [30]	To explore the experiences of adults following a sport-related knee injury and their attitudes	N/A	Individual semi-structured interviews	Inductive thematic analysis	UK	Amateur sports participants	λ Rugby λ Netball λ Football λ Roller derby	λ Local level λ National level	Acute knee injury	13 (7/6)	27 ± 4.6	The timing of retirement from sports or the prospect of no longer participating in sports is associated with changes in athletic identity.
Zavattaro, 2014 [69]	To use autoethnography to explore notions of self-identity formation and projection	λ Constructivist λ Poststructural	Autoethnogr-aphy	λ Narrative analysis λ Reflexive analysis	USA	Amateur sports participants	Outdoor activity	Amateur level	λ Anterior cruciate ligament torn λ Cartilage torn λ Quadriceps muscle torn λ Posterior cruciate ligament sprained	1 (0/1)	N/A	There is an association between sustaining a sports injury and athletic identity among amateur sports participants.
Zurek et al., 2022 [80]	To better understand the coping strategies and to identify factors that promote or hinder the successful adjustment of elite athletes after spinal cord injury	Descriptive-qualitative framework	Individual semi-structured interviews	λ Content analysis λ Thematic analysis	Poland	Athletes	λ Ski jumping λ Karate λ Rugby λ Mountain Biking λ Motocross λ Judo λ Speedway λ Bicycle Motocross	National level	Spinal cord injury	8 (6/2)	36.75 ± 8.08	Loss of functional ability is not necessarily associated with a loss of athletic identity.
Zwolski et al., 2024 [70]	To explore self-perceived changes in athlete journey trajectory, or shifts, after ACLR that facilitate or hinder physical activity participation among youth	Interpretive phenomenol-ogical methodology	Individual semi-structured interviews	λ Thematic analysis λ Constant comparison method	USA	Athletes	λ Volleyball λ Basketball λ Track and field λ Lacrosse λ Soccer, λ Swimming λ Football	High school level	ACL injury	10 (4/6)	Median (range) = 20.5 (18–28)	Among adolescent athletes, ACLR is associated with variations in the strength of athletic identity.

*ACLR* Anterior cruciate ligament reconstruction, *ACL* Anterior cruciate ligament, *PCL* Posterior cruciate ligament

**Fig. 2:**
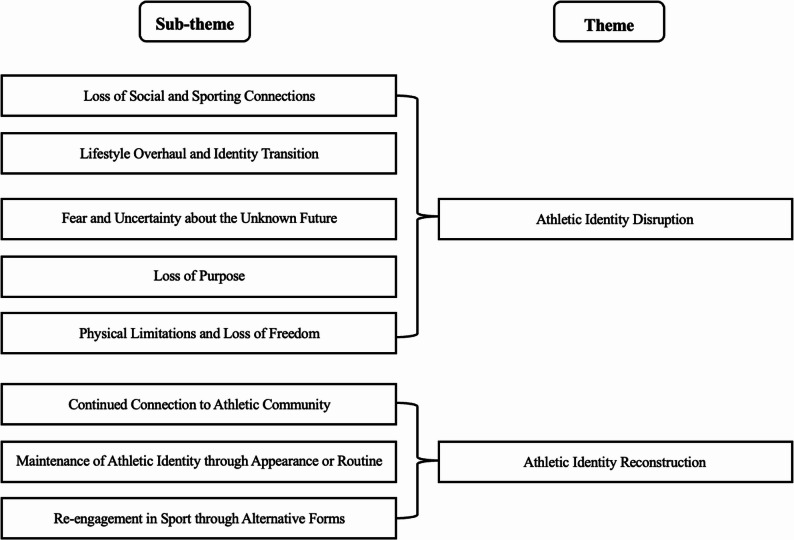
Thematic structure of the synthesized qualitative findings

A thematic synthesis of the included studies is provided in Appendix 1, outlining the major themes and subthemes identified during analysis. In addition to the table, a corresponding figure (see Fig. 2) is provided below to visually illustrate the hierarchical structure of the themes and subthemes.

### Confidence assessment

Confidence in the synthesized qualitative findings was evaluated using the ConQual approach [53]. This method considers two core components (i.e., dependability and credibility) to derive an overall ConQual score, representing the level of confidence in each synthesized finding. The dependability assessment results for each included study are presented in Table 3.

**Table 3:** Dependability Assessment of Included Studies (Alphabetical Order)

Author(s) and Year	Q1	Q2	Q3	Q4	Q5	Dependability Ranking
Borg et al., 2021 [60]	Y	Y	Y	Y	Y	Unchanged
Caron et al., 2017 [61]	Y	Y	Y	Y	N	Unchanged
Caron et al., 2021 [81]	Y	Y	Y	Y	Y	Unchanged
Cassilo and Sanderson, 2019 [62]	Y	Y	Y	Y	N	Unchanged
Crawford et al., 2014 [77	Y	Y	Y	Y	Y	Unchanged
Dean, 2018	Y	Y	Y	Y	Y	Unchanged
Douglas et al., 2024 [64]	Y	Y	Y	Y	N	Unchanged
Ezzat et al., 2018 [75]	Y	Y	Y	Y	Y	Unchanged
Hammer et al., 2019 [73]	Y	Y	Y	Y	Y	Unchanged
Hänninen and Pohjola, 2023 [65]	Y	Y	Y	Y	Y	Unchanged
Hawkins et al., 2014 [71]	Y	Y	Y	Y	N	Unchanged
Hockey, 2005 [76]	Y	Y	Y	Y	N	Unchanged
Karlström et al., 2022 [66]	Y	Y	Y	Y	Y	Unchanged
Kavanagh, 2012 [78]	Y	Y	Y	Y	N	Unchanged
Lisee et al., 2020 [67]	Y	Y	Y	Y	Y	Unchanged
Little et al., 2023 [68]	Y	Y	Y	Y	Y	Unchanged
Murray et al., 2022 [74]	Y	Y	Y	Y	N	Unchanged
Perrier et al., 2014 [79]	Y	Y	Y	Y	Y	Unchanged
Seguin and Culver, 2022 [31]	Y	Y	Y	Y	N	Unchanged
Sparkes and Smith, 2002 [72]	Y	Y	Y	Y	Y	Unchanged
Watkins et al., 2020 [30]	Y	Y	Y	Y	N	Unchanged
Zavattaro, 2014 [69]	Y	Y	Y	Y	Y	Unchanged
Zurek et al., 2022 [80]	Y	Y	Y	Y	Y	Unchanged
Zwolski et al., 2024 [70]	Y	Y	Y	Y	N	Unchanged

Q1 = Is there congruity between the research methodology and the research question or objectives? Q2 = Is there congruity between the research methodology and the methods used to collect data? Q3 = Is there congruity between the research methodology and the representation and analysis of data? Q4 = Is there a statement locating the researcher culturally or theoretically? Q5 = Is the influence of the researcher on the research, and vice-versa, addressed? Y = Y; No = N

The summary of findings and their associated confidence ratings are presented in Table 4.

**Table 4:** ConQual summary of findings

Systematic review title: Athletic Identity and Sport Injury: A Systematic Review and Meta-Aggregation									
Population: Athletes who sustained sports-related injuries									
Phenomena of interest: Status of athletic identity following sports-related injuries									
Context: Athletes currently experiencing or having a history of sports-related injuries									
Synthesized Finding	Categories						Dependability	Credibility	ConQual Score
	Name of Categories	Type of Research	Dependability	Credibility		ConQual Score			
Athletic Identity Disruption (*n* = 17)	Loss of Social and Sporting Connections	4 Descriptive Qualitative, 1 Narrative, 1 Phenomenology	Unchanged	10U, 8 C	Downgrade 1 Level	Moderate	Unchanged	Moderate	Moderate
	Lifestyle Overhaul and Identity Transition	2 Descriptive Qualitative, 1 Narrative, 1 IPA	Unchanged	8U, 5 C	Downgrade 1 Level	Moderate			
	Fear and Uncertainty about the Unknown Future	1 Descriptive Qualitative, 1 Narrative, 1 IPA	Unchanged	8U, 2 C	Downgrade 1 Level	Moderate			
	Loss of Purpose	1 Descriptive Qualitative, 1 IPA	Unchanged	2U, 1 C	Downgrade 1 Level	Moderate			
	Physical Limitations and Loss of Freedom	1 Descriptive Qualitative, 1 Autoethnography	Unchanged	7U	Unchanged	High			
Athletic Identity Reconstruction (*n* = 7)	Continued Connection to Athletic Community	2 Descriptive Qualitative, 1 Grounded Theory	Unchanged	7U, 5 C	Downgrade 1 Level	Moderate	Unchanged	Moderate	Moderate
	Maintenance of Athletic Identity through Appearance or Routine	1 Autoethnography	Unchanged	1U, 1 C	Downgrade 1 Level	Moderate			
	Re-engagement in Sport through Alternative Forms	2 Narrative, 1 Phenomenology	Unchanged	7U, 9 C	Downgrade 1 Level	Moderate			

*U* Unequivocal evidence, *C* Credible evidence, *IPA* Interpretative phenomenological analysis

A meta-aggregation of the findings produced two major themes: (1) athletic identity disruption and (2) athletic identity reconstruction. These themes capture shared experiences and perceptions across studies and form the basis for ConQual confidence assessments. Each theme is described in detail below, accompanied by illustrative quotes and context from the primary studies.

#### Athletic identity disruption

This theme includes 17 articles, all of which highlight the disruption of athletic identity following a sports injury. Seven of these studies specifically focus on traumatic brain injuries, such as concussions [31, 60–65].

In his autobiographical reflection on concussion recovery, Dean [63] described experiencing emotional struggles and a gradual loss of his student-athlete status due to reduced participation in sport. Similarly, Borg et al. [60], in interviews with athletes who had suffered concussions, found that participants experienced a loss of identity accompanied by negative emotions such as helplessness, fear, and anxiety. In Caron et al. [61], these athletes likened their mental state to that of an orchid: fragile, lonely, and fearful, and believed that their identity diminished accordingly. Hänninen and Pohjola [65], in their analysis of autobiographies of an ice hockey player who sustained traumatic brain injuries, reported: “For my whole life, I had constructed my identity on the basis of being an ice hockey player, and its termination like this would mean the collapse of my world” (p. 30). Participants in Douglas et al. [64] similarly indicated that concussions led them to lose part of their identity. In a theory-driven deductive analysis of public online support groups for athletes with concussion injuries, Cassilo and Sanderson [62] found comparable expressions of identity disruption: “Soccer was my life, it defined me as a person. Losing soccer was one of the hardest things I have ever had to go through” (p. 686). Likewise, Seguin and Culver [31] found that ice hockey players reported that following a concussion, not only their hockey life but even their overall sense of self appeared to disappear along with their athletic identity. These qualitative studies, employing diverse data analysis (e.g., Interpretative Phenomenological Analysis, Narrative Construct, Colaizzi Method, Thematic Narrative Analysis, Theory-driven Deductive Analysis), collectively demonstrate that concussion injuries pose a significant threat to athletes’ sense of identity.

In addition to studies on concussions, several others have explored the disruption of athletic identity following injuries such as knee and spinal cord injuries. Notably, anterior cruciate ligament (ACL) injuries are a common focus [30, 66–70], as well as spinal cord injuries [71, 72]. For example, Karlström et al. [66], in a series of individual semi-structured interviews with ACL-injured athletes, reported one participant stating, “It was like all of me and my entire identity. like disappeared. ” (p. 6). Similarly, Little et al. [68] found that athletes expressed a profound shift in self-perception after ACL injury: “I wasn’t able to be that sporty person and always be out training, playing sport. That whole perception of me being the sporty person, yeah, it just kind of went out the window” (p. 7).

Watkins et al. [30], also using individual semi-structured interviews with amateur athletes recovering from acute knee injuries, found that both the time away from sport and the uncertainty about returning significantly impacted athletes’ sense of identity. One participant described the experience as a difficult lifestyle shift that brought considerable emotional challenges, highlighting how injury disrupted their established athletic role.

Beyond knee and spinal cord injuries, other studies have documented similar identity disruptions among athletes with a range of injuries, including brachial plexus injury, visual impairment, neck injury, broken leg, and shoulder injury [73, 74]. For example, in their interviews with paratriathletes who became disabled due to injury, Hammer et al. [73] found that following the injury that caused their disability, athletes reported losing their life purpose and sense of meaning, as well as their athletic identity. Similarly, in an interpretative phenomenological analysis of elite male rugby union players, Murray et al. [74] found that following injury, athletes experienced feelings of demoralization and a sense of losing their identity. Collectively, these findings suggest that a wide variety of sports-related injuries can result in the disruption of athletic identity.

#### Athletic identity reconstruction

This theme includes seven studies, all showing that individuals reestablish their athletic identity through various means, following a sports injury. In studies by Ezzat et al. [75] and Hockey [76], participants reported that nutritional practices and rehabilitation training supported the recovery of their athletic identity. Similarly, athletes who acquired disabilities from injuries [77–79] often maintained their identity by adapting their activities or using special equipment (e.g., wheelchair).

Moreover, Zurek et al. [80] and Caron et al. [81] found that some injured athletes stayed engaged in the sports world through roles such as coaching, event organizing, or leading foundations, strategies that helped preserve and rebuild their athletic identity. A participant in Zurek et al.’s study [80], for instance, described continuing to attend races to support other athletes, illustrating how maintaining involvement in sport—even in non-performance roles—helped preserve their sense of being an athlete. Similarly, Caron et al. [81], in their narrative analysis, found that injured athletes maintained continued connection to the athletic community by assuming dual roles as team managers and student assistant coaches.

These findings suggest that athletic identity is not necessarily lost after injury but rather can be redefined and sustained through adaptive strategies and continued engagement with sport. Such efforts reflect the resilience of athletes in negotiating their identities post-injury, highlighting the dynamic and reconstructive nature of athletic identity.

## Discussion

This review aimed to synthesize qualitative research evidence on athletic identity following sports injuries. After reviewing and analyzing 24 qualitative studies, we identified two overarching analytical themes. One theme highlights the threat of identity disruption that many athletes experience during the recovery process. In contrast, the other theme supports findings from previous quantitative research [38], suggesting that athletic identity can be maintained after a sports injury. These two themes are discussed in turn.

First, one key theme that emerged from our synthesis is the experience of identity disruption. Specifically, some athletes face the challenge of losing their athletic identity following a sports injury. This disruption is shaped by a range of factors, including fear of not returning to sport [30, 61, 71], perceived changes in physical functionality [69, 73, 74], undergoing surgery [70], prolonged absence from competition [60], diminished social life and relationships [63, 66], the erosion of their athletic image in the eyes of others [68, 72], psychological unpreparedness to return [68], and medical diagnoses or recommendations [63, 65].

These factors help explain why athletes who maintained their athletic identity during the COVID-19 pandemic, despite limited access to training and competition, may not have experienced the same level of identity disruption [2, 25]. Wadey [29] notes that when injured, athletes’ lived experiences often conflict with the dominant cultural narrative of performance, contributing to threats to their athletic identity. This risk is especially pronounced among athletes who regard their athletic identity as their primary social identity, making them more vulnerable to identity disruption after an injury [31, 60, 65, 66, 72, 73].

Moreover, a strong athletic identity is closely linked to an individual’s sense of life meaning [82]. However, when athletic identity becomes the sole or dominant source of meaning, it can pose significant risks to mental health and overall well-being [82, 83], especially during transitions such as injury or retirement [84, 85]. In light of these risks, stakeholders in the sporting environment are encouraged to educate athletes about the dangers of overidentifying with the athlete role and the psychological consequences that may arise from identity disruption [86]. Supporting athletes in adopting a “multidimensional perspective” and fostering strong social networks is essential for resilience [57, 82]. As illustrated by Seguin and Culver [31] in their case study of Rachel, an ice hockey player who sustained a concussion: “She had to reshape her whole identity beyond that of an athlete. Social disconnect and these alien-like feelings were her new normal, and she had to find a way to live with that” (p. 13).

Social connections, particularly with teammates, play a critical role in mitigating the impact of identity disruption. Graupensperger et al. [25] highlighted that social support helps explain variations in athletic identity among student-athletes during the COVID-19 pandemic. Similarly, teammates’ prosocial behaviors have been shown to enhance identification with the team [87]. Liu and Noh [88], in a survey of injured athletes, found that perceived social support can contribute to the restoration of athletic identity. Accordingly, injured athletes who are temporarily disconnected from sport may be able to preserve or rebuild their athletic identity by maintaining close relationships with teammates and coaches or by remaining involved through roles such as assistant coaches [89]. In addition, athletes can be encouraged to cultivate new identities, gain fresh experiences, and develop interests or skills outside of sports [14, 90].

From a psychological intervention standpoint, sport psychologists can implement strategies, when appropriate, such as aerobic exercise programs, emotion-focused therapy, or even emerging methods like psychedelic-assisted therapy which is a form of mental health treatment that combines the controlled use of psychedelic substances with psychotherapy to help treat various psychological conditions, to support the emotional and identity recovery of injured athletes [28, 91, 92].

Second, the other key theme is athletic identity reconstruction. Across the included studies, many athletes were found to engage in various coping strategies to preserve or reconstruct their athletic identity. These strategies included taking on new roles such as event volunteer [80], team manager or assistant coach [81], referee [75], or participating in a different sport [79]. Consistent with the findings from Renton et al.’s quantitative review [38], these results suggest that athletic identity may remain relatively stable despite injury.

Furthermore, athletes who sustained severe or disabling injuries were often able to rebuild their athletic identity through involvement in para-sports competitions [77–79] and by adopting adapted social roles such as coach, advocate, or sports committee member [1]. These forms of continued engagement represent meaningful ways for athletes to maintain or reshape their sense of athletic identity in the face of significant physical and psychological challenges.

It is important to note that several studies included participants from non-athlete populations, such as amateur sports participants [30, 66, 68, 69, 75, 79]. Compared to competitive athletes, non-athletes tend to report lower levels of athletic identity [93] and experience lower levels of anxiety after injury [94]. However, Mohrsen et al. [27] suggest that for non-athletes, being forced to stop or reduce physical activity due to injury may result in the loss of a valued social identity connected to sport. Moreover, non-athletes are more likely to permanently relinquish their athletic identity after an injury [79].

Seo and Reifsteck [95] emphasize that athletic identity and exerciser identity differ significantly in terms of concept, motivation, and cultural context. This distinction is especially relevant in quantitative research, where measurement tools such as the AIMS and AIMS-3G were specifically designed for athletic populations [4, 43]. The applicability and sensitivity of these instruments for non-athlete groups remain uncertain, and their inappropriate use could lead to misleading conclusions.

### Research limitations and future recommendations

There are several limitations to acknowledge for this review. First, this review was the exclusion of mixed-methods research. Mixed-methods studies, which integrate both qualitative and quantitative approaches, offer a more comprehensive perspective and can help bridge the differences observed between these two methodologies. Their inclusion could provide a deeper understanding of how athletic identity evolves following sports injuries and helps explain inconsistencies across findings.

Second, it is important to note that the conclusions drawn in this review are primarily based on studies that relied exclusively on retrospective self-reports from athletes. Such methods are subject to recall bias and may be influenced by the participants’ current psychological state or hindsight interpretation of past events. Future research would benefit from incorporating mixed-methods studies to explore the relationship between changes in athletic identity and psychological variables, such as readiness to return to sport and adherence to rehabilitation protocols [8, 35].

Third, this review included only English-language publications. This approach may introduce language bias, as studies conducted in non-English-speaking regions were not captured, thereby reducing the cultural and contextual diversity of perspectives represented in the synthesis. Consequently, the generalizability of findings—particularly to non-elite or community-level athletes from diverse cultural backgrounds—is limited.

Fourth, although the search terms in this review were informed by previous systematic reviews on sports injury and athletic identity [38, 39], half of the included studies (*n* = 12) were identified through manual searches. This suggests that the initial database search strategy may have had limited reach and may have missed relevant literature. Given these limitations, we strongly recommend that future systematic reviews on athletic identity include comprehensive manual searches and expand search terms to encompass broader concepts related to athletic identity post-injury, such as psychological responses, identity transition, and posttraumatic growth.

Fifth, as stated in the first exclusion criterion of this review, an athlete’s social identity is not necessarily equivalent to their athletic identity [2]. For example, among disabled athletes, even those who invest significant time and effort in training and competition may not regard their athletic identity as their primary social identity [2]. Thus, future quantitative research should clearly distinguish between athlete and non-athlete groups, such as exercisers, through well-defined inclusion and exclusion criteria, in order to improve the sensitivity and appropriateness of the athletic identity scales. Likewise, qualitative studies should explicitly clarify whether their focus is on athletic identity or broader forms of social identity. Such efforts will contribute to enhancing the overall validity and reliability of research in this field.

Sixth, it is important to acknowledge the inherent subjectivity involved in assessing the quality and confidence of qualitative studies. Although this review employed established tools such as the MMAT, JBI, and ConQual approach to enhance transparency and rigor, the interpretation of methodological criteria and the weighting of evidence inevitably involve researcher judgment. This subjectivity may have resulted in the overestimation or underestimation of the contribution of certain studies, particularly when evaluating constructs such as credibility, dependability, and the depth of qualitative insight. To mitigate this limitation, assessments were conducted systematically and guided by predefined criteria. However, future reviews may benefit from multiple independent reviewers, consensus procedures, or complementary appraisal frameworks to further strengthen reliability. Recognizing these constraints is essential when interpreting the synthesized findings and their implications for athletic identity and injury experiences.

Lastly, an important consideration is the potential influence of cultural background on athletes’ experiences of identity and injury. Although the synthesized findings may appear relatively homogeneous, this consistency likely reflects the predominance of studies conducted in Western, individualistic cultural contexts. Cultural norms shape how athletes construct their identities, interpret injury, and engage with support systems. For example, in collectivist cultures, athletic identity may be closely tied to family expectations, community reputation, or national pride, which could intensify feelings of guilt, pressure, or obligation during injury. Conversely, in cultures that emphasize individual autonomy, athletes may experience injury as a personal setback that threatens self-achievement rather than social belonging. Cultural values also influence how athletes interpret injury, seek support, and reconstruct their sense of self. For example, athletes embedded in religious cultural contexts may draw on their faith as a source of meaning, coping, and psychological adjustment throughout the rehabilitation process [96]. Because most included studies did not explicitly analyze cultural dimensions, the extent to which cultural background shapes identity disruption, coping strategies, or post-injury adjustment remains underexplored. Therefore, the findings should be interpreted with caution, and future qualitative studies should incorporate culturally diverse samples and examine cultural frameworks more explicitly to deepen understanding of how athletic identity and injury are experienced across different sociocultural contexts.

### Practical implications

One of the primary aims of meta-aggregation is to generate practical guidance for application in real-world contexts. By synthesizing qualitative evidence on changes in athletic identity following sports injuries, this review highlights the need for sport psychologists to recognize and address identity-related challenges faced by injured athletes.

Firstly, practitioners (e.g., coaches and sport psychologists) should inform athletes about the risks of identity disruption following injury and help them recognize that changes in how they view themselves are a normal part of the recovery process [86]. By encouraging athletes to articulate their personal narratives around injury, practitioners can support the development of a more flexible and balanced sense of self, reducing the likelihood that athletes become overly defined by their injured status. This approach is particularly valuable for athletes with a highly exclusive athletic identity, youth athletes who are still forming their sense of self, and elite performers whose careers depend heavily on sport success.

Secondly, for coaches, the findings suggest the importance of creating a supportive environment that validates athletes’ emotional experiences and reinforces their value to the team beyond physical performance. Coaches can facilitate positive outcomes by maintaining communication during rehabilitation, offering meaningful non-physical roles (e.g., leadership, mentoring), and collaborating with sport psychologists to integrate identity-strengthening strategies into training. These practical applications can help injured athletes cultivate a more adaptive identity, maintain motivation throughout rehabilitation, and ultimately achieve a healthier and more resilient return to sport.

In addition, given the limited availability of interventions specifically targeting post-injury athletic identity loss, practitioners should consider adapting interventions designed for psychological recovery after injury, such as meditation-based interventions [97]. Given that athletic identity is associated with emotional responses and adherence during rehabilitation following injury [31], such efforts may be adaptive for some individuals in certain situations.

Thirdly, for policymakers, the evidence suggests a need for policies that support structured psychological services addressing athletic identity challenges, including post-injury and post-retirement transitions. Such initiatives may include providing accessible mental health resources, offering education on identity-related risks, and promoting social identity development beyond athletic roles, for example, by offering injured athletes part-time opportunities as assistant coaches or team managers to facilitate their active engagement with the team during the recovery period. Such measures may help protect the long-term mental health and well-being of athletes at all levels of participation.

Lastly, for athletes who retire due to injury, sports psychologists play a crucial role in facilitating post-retirement life planning and encouraging the development of multiple social identities, such as taking on roles as coaches, team managers, or members of sports committees and organizations beyond sports [2, 80]. Such efforts can foster a sense of purpose and continuity, thereby supporting mental well-being during the transition out of athletic careers.

## Conclusion

This systematic review synthesized qualitative studies exploring how athletic identity is experienced following sports injuries. The findings suggest that some athletes may experience disruption of their athletic identity post-injury, while others are able to preserve or reconstruct it through adaptive coping strategies, such as engaging with sport in new roles like assistant coach, team manager, or committee member. Future research should consider more diverse study designs, such as longitudinal and mixed-methods studies, and further investigate how cultural, social, and contextual factors influence athletes’ experiences of identity disruption and reconstruction.

## Supplementary Information

Supplementary Material 1.

## Data Availability

Data available on request from the authors. The data that support the findings of this review are available from the corresponding author, upon reasonable request.
