# Supplementary material for: Athletic identity and sport injury: a systematic review and meta-aggregation
Source: BMC Psychol. 2025 Dec 23;14:111. doi: 10.1186/s40359-025-03902-7 (PMC12837347; doi:10.1186/s40359-025-03902-7)
Supplement: Supplementary file 1 — Supplementary Material 1. [file 40359_2025_3902_MOESM1_ESM.docx]

Appendix 1

Synthesised Findings

| **Author (s) and Year** | **Findings** | **Categories** | **Synthesized Findings** |
| --- | --- | --- | --- |
| Seguin and Culver, 2022 | - She was a hockey player. Once that aspect of herself slowly started disappearing, her hockey family seemed to disappear. (C)^*^ - That’s when she realized it wasn’t just her hockey life that was disappearing, it was her whole life. (C) - She didn’t feel normal and she felt like she was losing her sense of who she was. (C) | Sub-theme (1): Loss of Social and Sporting Connections | Theme (1): Athletic Identity Disruption |
| Sparkes and Smith, 2002 | - Associated with and intimately connected to the loss of the masculine self for Matthew, Mark, Craig, and Eamonn was the loss of their social, personal, and corporeal athletic identities. (C) - I think, because rugby played an important part in my life, not only the playing but socializing as well, people recognized me as a sportsman. It was a big part of my life and your friends are from the [rugby] club, so of course you come to think of yourself as a sportsman, even though you know you are not going to play for England or anything....Now, well, I miss that side of my life. I miss being a sportsman and everything that goes with it. It was a big part of me. Now, because of the injury, I’m really no one, not who I really am. (U) | Sub-theme (1): Loss of Social and Sporting Connections | Theme (1): Athletic Identity Disruption |
| Dean, 2019 | - From the moment I was diagnosed with a concussion, I began to (re)negotiate my athletic identity. (U) - Emotions such as anger, social isolation, depression, and anxiety were constantly seeping into my daily life both publicly and privately, while my social status as a student-athlete was slowly being stripped from me due to my lack of participation and involvement within the sporting culture. (U) | Sub-theme (1): Loss of Social and Sporting Connections | Theme (1): Athletic Identity Disruption |
| Cassilo and Sanderson, 2019 | - Still others framed loss around their athletic identity. (C) - I am still not cleared for gym or sports. I am now 13 years old and can’t play hockey again and am really not supposed to do any contact sports ever again. (U) - The hardest part of not being able to play is watching everyone play a sport you love. (U) - Soccer was my life; it defined me as a person. Losing soccer was one of the hardest things I have ever had to go through. (U) - I soon came to the conclusion that this was the end for me. As I unlaced my shoes and ankle braces for the last time ever, tears ran down my face. (U) - I was done with my life of soccer as well as my new life of lacrosse. It was the worst news I have ever gotten. The thought of never being able to play soccer again after playing for 11 to 12 years broke my heart. (U) | Sub-theme (1): Loss of Social and Sporting Connections | Theme (1): Athletic Identity Disruption |
| Douglas et al., 2024 | - Athletes discussed feeling as though they lost a piece of their identity after sustaining their concussion. (C) - I would just sit here... start crying because ...I can’t play my favorite sport. Like I can’t do what I love.” (U) | Sub-theme (1): Loss of Social and Sporting Connections | Theme (1): Athletic Identity Disruption |
| Karlström et al., 2022 | - Identity challenges implied a sensed ‘loss of identity’, as the injury not only affected the knee but the whole person and personality, often tied to the sacrifices made to manage overall life. (C) - It was like all of me and my entire identity... like disappeared... (U) - Experiences of being changed in the eyes of others were mainly connected to the inability to engage in sports and all the physical activities that had formed part of their (former) social life and relationships. (C) | Sub-theme (1): Loss of Social and Sporting Connections | Theme (1): Athletic Identity Disruption |
| Watkins et al., 2020 | - Furthermore, time out of their sport, or the prospect of not returning to the sport, had affected their athletic identity and this had repercussions for their broader quality of life. (C) - It certainly was very difficult, I suffered with a few mental health concerns, because it was that dedication, and that dedication to sport that obviously being told you can’t do it anymore; it was a difficult change because it was a whole lifestyle overhaul for me. (U) | Sub-theme (2): Lifestyle Overhaul and Identity Transition | Theme (1): Athletic Identity Disruption |
| Hänninen and Pohjola, 2023 | - After their athletic identities had fully developed, both Kovanen and Hyysalo encountered devastating setbacks: Kovanen was violently tackled, and Hyysalo landed on his head after a jump. (C) - “I felt like that five-year boy”—but something had changed: he could not skate. (U) - I stared at the ice that had suddenly turned alien to me. (U) - It was only when his neurologist wrote in a statement that his “possibilities to return to the profession of ice hockey player are becoming slender ” that he started to realize that his career was over. (U) - For my whole life, I had constructed my identity on the basis of being an ice hockey player and its termination like this would mean the collapse of my world. (U) - It didn’t even occur to me that I couldn’t ski anymore. (U) | Sub-theme (2): Lifestyle Overhaul and Identity Transition | Theme (1): Athletic Identity Disruption |
| Zwolski et al., 2024 | - The participants expressed changes in athlete identity, with ACLR and life transitions reported as perceived contributing factors. (C) - In the years following ACLR, the strength of athlete identity was perceived to lessen, yet remain ingrained despite transition away from competitive sport. (C) - I mean (laughs), it’s kind of just ingrained in who I am, in my DNA at this point; Uh, you know, just...I wouldn’t say I’m an athlete. I’m a, I guess an adult that (laughs) tries to stay fit and wants to look okay if I take my shirt off at the beach (laughs). (U) | Sub-theme (2): Lifestyle Overhaul and Identity Transition | Theme (1): Athletic Identity Disruption |
| Lisee et al., 2020 | - Females encountered a more noticeable daily disruption of their athletic identity that may have led to more intense mood shifts associated with psychological distress. (C) - Basketball was hard cause all I did was sit there and kept books at the games, and track was hard cause of watching high jump. High jump is my passion, so watching that, it was hard and then… they had a new kid that jumped, and he beat my PR [personal record] at second jump that he did, so that was hard. (U) | Sub-theme (2): Lifestyle Overhaul and Identity Transition | Theme (1): Athletic Identity Disruption |
| Caron et al., 2017 | - There was an orchid in my room. It was starting to lose some of its petals because I stopped watering it. The orchid’s state matched my mood: It was rather frail, lonely, and afraid. I was afraid of the next day, afraid of tomorrow.... It was a fear of the unknown. (U) - In many ways, like the orchid, Daphnée’s identity of who she was becoming was lost. Her future stories of being a successful volleyball player and a thriving academic took a back seat to an uncertain and unknown future. (C) | Sub-theme (3): Fear and Uncertainty about the Unknown Future | Theme (1): Athletic Identity Disruption |
| Borg et al., 2021 | - It was more difficult because I felt I was losing my identity rather than because I couldn’t finish the season because it was practically over and it wasn’t mathematically possible for us to win the league. (U) - While they identified football as giving them satisfaction, participants experienced loss of identity, causing negative feelings including helplessness, fear, and anxiety when they sustained an injury. (C) - I didn’t know who I was anymore. Who’s [Tom]? Before people would say, “He plays football”. (U) | Sub-theme (3): Fear and Uncertainty about the Unknown Future | Theme (1): Athletic Identity Disruption |
| Little et al., 2023 | - Participants discussed a change in self-identity, sporting participation, and self-belief which contributed to fear. They spoke about losing their sense of athletic identity. (U) - I wasn’t able to be that sporty person and always be out training, playing sport . . . That whole perception of me being the sporty person, yeah, it just kind of went out the window. (U) - I can’t play basketball anymore because of my knees, and I’m like, I’m 25 . . . it’s disabling a little bit. Physically, I can do those things, but it’s the mental factor that’s stopping me. It’s frustrating, the fear is what’s stopping me. (U) - I was very competitive at hockey, I was playing a good grade, and I just wouldn’t be able to get back to that. (U) - Everyone said I should have been right to play, so I did go back to try and play. I was just like, ‘No, I can’t. There’s something wrong, so I can’t keep playing’. . . . I just never went and tried playing again. (U) | Sub-theme (3): Fear and Uncertainty about the Unknown Future | Theme (1): Athletic Identity Disruption |
| Hammer et al., 2019 | - Many participants also reported that acquiring a disability resulted in feeling like they lost their identity. (C) - Pre-injury my purpose in life was to be a phenomenal athlete. And then, immediately post-injury, there was a loss of purpose. To be honest with you, I felt like I didn’t know what I was here for. Everything was stripped from me, and all of those things I thought I was supposed to be before were gone. (U) | Sub-theme (4): Loss of Purpose | Theme (1): Athletic Identity Disruption |
| Murray et al., 2022 | - .... it was almost like a loss of identity, now I’m just.... just this battler...just a small guy who can pass... but, [names another player] already does that so what am I gonna be good for? ... well, nothing... yeah... like a little loss of identity... (U) | Sub-theme (4): Loss of Purpose | Theme (1): Athletic Identity Disruption |
| Hawkins et al., 2014 | - It has completely changed my life. I was going off to Bournemouth University beforehand, but because of my SCI, it changed my whole direction in life. (U) - After my accident, I had to re-train; unfortunately, office work suited my condition the best for both access and conditions. (U) - It makes me worry about the future and it does make me miss my old life. (U) - I do get down a lot because I do miss my old life. (U) - I have lost lots of my freedom and do miss being spontaneous. (U) - I lost my freedom to do what I like. (U) | Sub-theme (5): Physical Limitations and Loss of Freedom | Theme (1): Athletic Identity Disruption |
| Zavattaro, 2014 | I lost my athletic identity. I went from being physically active to passively sitting with small hand weights to feel like I was doing something for my health benefit. (U) | Sub-theme (5): Physical Limitations and Loss of Freedom | Theme (1): Athletic Identity Disruption |
| Zurek et al., 2022 | - Despite the fitness loss, they did not lose their identity. In accordance with their vision of themselves, they set new goals and chose ways to achieve them. (C) - My identity has always been of an athlete and so now not to have that as my identity was frightening really scary during that time. (U) - . . . in fact life didn’t end that you can still do sports, maybe a little bit in a different form because unfortunately for that you need some kind of special equipment, but it was possible. Well there was some kind of a signal that I was saying that he can continue to do that skiing that I used to do before the accident, I’m still doing it, and that’s how it went. (U) - . . . in fact life didn’t end that you can still do sports, maybe a little bit in a different form because unfortunately for that you need some kind of special equipment, but it was possible. (U) - I still sometimes take part in, appear at the start or finish line of, for the benefit of the earth, or marathons, also supporting runners, because I myself enjoyed running before the accident. And after the accident, from friend to friend, it so happened that I also support the runners. Also at some stops, finish line or start I am with them. I don’t run marathons with them because my arms would fall off, but at least this way I spend time with them. (U) | Sub-theme (1): Continued Connection to Athletic Community | Theme (2): Athletic Identity Reconstruction |
| Caron et al., 2021 | - He offered her the dual role of team manager and student assistant coach while she was unable to play. (C) - As Cassie’s role and identity were shifting as a result of the concussion, so, too, were her emotions. (C) | Sub-theme (1): Continued Connection to Athletic Community | Theme (2): Athletic Identity Reconstruction |
| Ezzat et al., 2018 | - As part of this change in athletic identity, participants discussed shifting from team sports to more individualized sports or engaging in new sport roles, such as coaching or refereeing. (C) - The switch from team sports more to individual kind of training and personal goals rather than like you know part of that sort of team political organization kind of thing and not like I don’t play for any club I haven’t played for any clubs or anything recently. I don’t know if I’ll go back to rugby just cause I don’t want to get injured again but, yeah, so I guess the idea of being playing on a team has switched now to just being athletic for myself. (U) - I am reffing basketball, I coach basketball at a high school, and I also play intramural basketball at the university. (U) - There were contrasting perspectives on the degree to which participants still saw themselves as athletes. (C) - I still consider myself an athlete cause, I keep myself in shape wise to do things, there’s not anything I cannot pick up and do. (U) | Sub-theme (1): Continued Connection to Athletic Community | Theme (2): Athletic Identity Reconstruction |
| Hockey, 2005 | - The continuation of this routine nutritional practice played a fundamental part in the maintenance of our embodied running identities. (C) - I noticed today that it’s 4 months since we have run. What’s interesting is that neither of us has put on any extra weight, so whilst at the moment we can’t run or even jog, we still look like distance runners. That helps because I can still see myself in the mirror and not someone else. I feel that would be even more difficult if I couldn’t see my proper self. I know I can’t run at the moment, I know I’m totally unfit for running, but it looks as if I am still running. That’s comforting because objectively I know when I start running again the experience will not be as hard as if I were carrying surplus poundage. More importantly, I feel I am still here. I can see my running self. So because I still look like I can run, the possibility is I will eventually. (U) | Sub-theme (2): Maintenance of Athletic Identity through Appearance or Routine | Theme (2): Athletic Identity Reconstruction |
| Perrier et al., 2014 | - These participants felt that although their present behavior was not in line with that of an athlete; an athletic identity could be restored once their future behavior changes. (C) - I got into a climbing harness and, you know, tied up, tied in, got my belay set up with my belay partner and just had at it, and I mean, I didn’t climb pretty. I didn’t climb well. I fell a couple of times, but I got to the top of a route and I had not felt that sense of elation since well before my accident. I mean, I distinctly remember that feeling. And every time I climb now, I still have that feeling, so I feel like that’s going in the right direction. (U) - I kind of think of myself in the same way. I mean, the disability does affect my identity, but it doesn’t affect it. I mean it is a part of who I am, clearly. There’s no getting around that. But it definitely doesn’t define me, it isn’t the predominant characteristic of who I am, which I guess can happen ... it’s affected my identity simply from the fact that I have to ask for help. But I mean we all ask for help for certain things anyways, right? Nobody gets through the day totally by themselves, no matter who you are. I don’t think it’s really impacted my identity all that much other than maybe the way some other people would initially react to me. (U) - I can’t say that I’m anywhere close to being someone who could be a competitive skier; however, every time that I’ve been out, I’ve seen an increase in the skill ... I see the growth of my skill and the growth of my confidence. I think that’s what, that’s what keeps me going back, and that’s what I really think an athlete is. Someone who is striving to do, do more and to improve their skill at whatever it is that they’re working on. (U) - As an example, Erin identified that while she uses different equipment, she is still competing and is therefore still an athlete. (C) | Sub-theme (3): Re-engagement in Sport through Alternative Forms | Theme (2): Athletic Identity Reconstruction |
| Kavanagh, 2012 | - For Lucy a momentous decision to take up sport again created the pathway for this personal rediscovery through physical activity. (C) - Through participation in wheelchair tennis, Lucy has been able to redesign an aspect of herself as an athlete creating a new athletic identity central to her rehabilitation. (C) | Sub-theme (3): Re-engagement in Sport through Alternative Forms | Theme (2): Athletic Identity Reconstruction |
| Crawford et al., 2014 | - Many participants experienced a forced new identity following the onset of SCI and were required to confront challenging life circumstances including learning how to travel in a wheelchair and/or complete daily activities. (C) - So then I lost my sexuality, the image that I was, everything was totally gone. I had to totally rebuild myself as a new person. (U) - Although some participants did not initially understand the significance of their injury, they described themselves as remaining “mentally tough” for their families and as time progressed, realized the outcomes associated with their injury and the impact on their identity. (C) - ... I also did not realize the more nuance things. Such as, you know, your identity being completely uh shattered more or less. (U) - Four participants highly involved in competitive sports also experienced a forced new athletic identity because they were unable to return to sport and practice or compete in the same manner they had prior to the SCI. (C) - Many commented about sport allowing them to re-establish their identity on a new basis after the occurrence of SCI. (C) - For one individual, the process of relearning a familiar sport proved challenging, but allowed the participant to re-establish his identity within the sport. (C) - … I love sports. They were a large part of my identity before my injury. It was definitely hard especially because I was such a good skier before. It definitely challenged me to like relearn some things that I was reluctant to learn. If you are really good at something and then you end up having to relearn something it is a huge blow to your self-esteem, at least it was for me. It was a way to re-identify and rediscover myself with sport. (U) - I always played contact sports growing up. You think you kind of lost that ability to do that type of thing. But when I found out about rugby and realized it was full contact, I was like even though I can’t walk and run around, I can still play full contact in a chair. And that was pretty cool for me knowing that aspect was still there. In high school, I was always the “athlete” that is just who I was. ... Knowing that part of my life was still there was huge for me. (U) | Sub-theme (3): Re-engagement in Sport through Alternative Forms | Theme (2): Athletic Identity Reconstruction |

**Note*: U = Unequivocal; C = Credible; TBI = traumatic brain injury; SCI = spinal cord injury; ACLR = anterior cruciate ligament reconstruction.
